# Regression of Left Ventricular Mass in Athletes Undergoing Complete Detraining Is Mediated by Decrease in Intracellular but Not Extracellular Compartments

**DOI:** 10.1161/CIRCIMAGING.119.009417

**Published:** 2019-09-11

**Authors:** Peter P. Swoboda, Pankaj Garg, Eylem Levelt, David A. Broadbent, Ashkun Zolfaghari-Nia, A. James R. Foley, Graham J. Fent, Pei G. Chew, Louise A. Brown, Christopher E. Saunderson, Erica Dall’Armellina, John P. Greenwood, Sven Plein

**Affiliations:** 1 Department of Cardiovascular Imaging Science, Leeds Institute of Cardiovascular and Metabolic Medicine, University of Leeds, United Kingdom (P.P.S., P.G., E.L., D.A.B., A.Z.-N., A.J.R.F., G.J.F., P.G.C., L.A.B., C.E.S., E.D., J.P.G., S.P.).; 2 Medical Physics and Engineering, Leeds Teaching Hospitals NHS Trust, United Kingdom (D.A.B.).

**Keywords:** athletes, magnetic resonance imaging, hypertrophy, sports

## Abstract

**Background:**

Athletic cardiac remodeling can occasionally be difficult to differentiate from pathological hypertrophy. Detraining is a commonly used diagnostic test to identify physiological hypertrophy, which can be diagnosed if hypertrophy regresses. We aimed to establish whether athletic cardiac remodeling assessed by cardiovascular magnetic resonance is mediated by changes in intracellular or extracellular compartments and whether this occurs by 1 or 3 months of detraining.

**Methods:**

Twenty-eight athletes about to embark on a period of forced detraining due to incidental limb bone fracture underwent clinical assessment, ECG, and contrast-enhanced cardiovascular magnetic resonance within a week of their injury and then 1 month and 3 months later.

**Results:**

After 1 month of detraining, there was reduction in left ventricular (LV) mass (130±28 to 121±25 g; *P*<0.0001), increase in native T1 (1225±30 to 1239±30 ms; *P*=0.02), and extracellular volume fraction (24.5±2.3% to 26.0±2.6%; *P*=0.0007) with no further changes by 3 months. The decrease in LV mass was mediated by a decrease in intracellular compartment volume (94±22 to 85±19 mL; *P*<0.0001) with no significant change in the extracellular compartment volume. High LV mass index, low native T1, and low extracellular volume fraction at baseline were all predictive of regression in LV mass in the first month.

**Conclusions:**

Regression of athletic LV hypertrophy can be detected after just 1 month of complete detraining and is mediated by a decrease in the intracellular myocardial compartment with no change in the extracellular compartment. Further studies are needed in athletes with overt and pathological hypertrophy to establish whether native T1 and extracellular volume fraction may complement electrocardiography, echocardiography, cardiopulmonary exercise testing, and genetic testing in predicting the outcome of detraining.

**Clinical Perspective**

Regular athletic training leads to physiological cardiac adaptation, namely biventricular dilatation and left ventricular hypertrophy, sometimes termed athlete’s heart. Cross-sectional studies using cardiovascular magnetic resonance T1 mapping imply that athletic left ventricular hypertrophy is mediated by an increase in the intracellular compartment (predominantly myocytes), with a relatively constant extracellular compartment (extracellular matrix and capillary vasculature). We studied athletes who were about to embark on a period of forced detraining due to incidental limb bone fracture within a week of their injury and then 1 month and 3 months later. On complete detraining, athletic left ventricular hypertrophy regressed within a month, which was mediated by decrease in the size of the cellular compartment with no change in the extracellular compartment. T1 mapping is a powerful tool to investigate mechanisms and reversibility of hypertrophy in athletes and may have a role in predicting the outcome of detraining. However, it remains to be validated in athletes with abnormal ECG and suspected cardiomyopathy.

## Introduction

The regular training that is required to participate in competitive sport leads to physiological cardiac adaptation, namely biventricular dilatation and left ventricular (LV) hypertrophy, sometimes termed athlete’s heart.^1^ LV hypertrophy that overlaps into the pathological range is relatively rare although it does occur more commonly in participants of certain sports such as rowing and cycling and in male and black athletes.^2^

Cardiovascular magnetic resonance (CMR) is commonly used in the assessment of athletes with LV hypertrophy because it allows both visualization of the LV in multiple imaging planes and detection of focal scar by late gadolinium enhancement (LGE) imaging. T1 mapping by CMR has been proposed to investigate cardiac tissue characteristics in athletes. Athletes have lower cardiac extracellular volume fraction (ECV) than sedentary controls, and the fittest athletes have the lowest ECV.^3,4^ These cross-sectional findings imply that athletic LV hypertrophy is mediated by an increase in the intracellular compartment (predominantly myocytes), with a relatively constant extracellular compartment (extracellular matrix and capillary vasculature). Conversely, areas of hypertrophy in hypertrophic cardiomyopathy (HCM) have increased ECV, and initial data suggest that this divergent pattern might be useful to differentiate it from athlete’s heart.^5^

Neither native T1 nor ECV has been validated histologically in athletic hypertrophy. However preclinical models of athlete’s heart have shown that increase in LV mass in exercise-trained rats is not associated with increase in collagen fraction, therefore, implying it is mediated by increase in myocyte mass.^6,7^

Detraining is a commonly used test to diagnose athletic remodeling, although data supporting its use are limited. After long-term cessation of training, regression of hypertrophy and dilatation occurs in 80% of athletes with cardiac dimensions outside the normal range.^8^ A minimum of 3 months of detraining is typically required to demonstrate regression of LV hypertrophy albeit with reduction in training rather than complete cessation.^9,10^ Compliance with detraining is often poor, and it is unpopular with athletes of all levels. There is, therefore, a need to improve the identification of athlete’s heart by noninvasive imaging and predict the outcome of detraining.

We hypothesized that in athletes who completely stop all forms of training, regression of LV hypertrophy occurs in 1 month and is mediated by a decrease in intracellular compartment volume. We aimed to investigate whether T1 mapping findings at baseline are predictive of the cardiac consequences of detraining.

## Methods

The data that support the findings of this study are available from the corresponding author on reasonable request. We prospectively recruited athletes presenting to the Emergency Department in Leeds Teaching Hospitals NHS Trust with a limb bone fracture for which they were advised to stop training for a minimum of 6 weeks. Only athletes aged 18 to 45 years who trained for >4 hours a week for >2 years were recruited. Exclusion criteria were any significant medical history, any regular medication, or self-reported use of anabolic steroids. Some athletes were able to recommence light training (fewer hours and lower intensity than preinjury levels) before their 3-month appointment, and this was not prohibited by the research team. The study was approved by the National Research Ethics Service (16/EM/0399), and all participants gave written informed consent.

Appointments occurred within a week of the injury, 1 month and 3 months later. At each appointment, athletes underwent clinical assessment, contrast-enhanced CMR, and 12-lead ECG. Twelve-lead ECG (MAC500; GE Medical Systems, Milwaukee, WI) was analyzed by 2 physicians blinded to clinical details according to international guidelines for ECG interpretation in athletes.^11^ LV mass was estimated from ECG by the Sokolow-Lyon product, the voltage sum of the greatest S wave in V_1/2_ and R wave in V_5/6_.^12^ A full blood count, for measurement of hematocrit, was taken at the time of intravenous cannulation before each CMR study.

### CMR Acquisition

Participants underwent CMR on a dedicated cardiovascular 3-Tesla Philips Achieva system equipped with a 32-channel coil and MultiTransmit technology. Data were acquired during breath holding at end expiration. Balanced steady-state free precession cine images covering the entire heart in the LV short axis were acquired before contrast administration (repetition time, 2.7 ms; echo time, 1.3 ms; matrix, 320×320; slice thickness, 10 mm with no gap; 30 cardiac phases).

T1 maps were acquired in 3 short-axis slices. Native T1 mapping used a breath-held modified Look-Locker inversion recovery acquisition (ECG triggered, 5 s (3 s) 3 s; single-shot, SENSE factor 2; prepulse delay, 350 ms; trigger delay set for end diastole [adaptive]; flip angle, 20°; matrix, 400×400; slice thickness, 10 mm; giving a reconstructed voxel size of 1.17×1.17 mm).

Gadobutrol (0.15 mmol/kg) was administered through an intravenous cannula with a 10-mL saline flush (Gadovist; Bayer Pharma, Berlin, Germany).

Tissue tagging by spatial modulation of magnetization (spatial resolution, 1.51×1.57×10 mm^3^; tag separation, 7 mm; ≥18 phases; repetition time, 5.8 ms; echo time, 3.5 ms; flip angle, 10°; typical temporal resolution, 55 ms) was acquired in the 3 short-axis slices.^13^

LGE in matching LV short-axis planes was performed >6 minutes after contrast administration. Typical parameters were repetition time, 3.7 ms; echo time, 2.0 ms; flip angle, 25^o^; matrix, 512×512; and slice thickness, 8 mm with 2 mm gap.

Post contrast T1 mapping was performed exactly 15 minutes after last contrast injection using 4 s (3 s) 3 s (3 s) 2 s modified Look-Locker inversion recovery acquisition with identical positioning and planning to the native T1 map.

### CMR Analysis

CMR data were assessed quantitatively using commercially available software blinded to detraining status (CVI42; Circle Cardiovascular Imaging, Inc, Calgary, Canada). Epicardial and endocardial borders were traced offline on the short-axis cine stack at end diastole and end systole to calculate LV and right ventricular (RV) end-diastolic volume (EDV), end-systolic volume, stroke volume, ejection fraction, and LV mass. Papillary muscles were excluded from all measurements. Indexed cardiac parameters were divided by body surface area calculated by the Mosteller equation at baseline.^14^ LGE imaging was analyzed visually to assess for the presence of scarring.

Pre- and postcontrast myocardial T1 values with a 3-parameter exponential fit with Look-Locker correction were measured from short-axis slices in the septum. Average measurements from the basal and mid ventricular slices were used. Data from the apical slice was not used because it was vulnerable to partial volume effects because of decreased wall thickness. ECV was calculated from native and postcontrast T1 times of myocardium and blood pool and hematocrit as reported previously.^15^

Intracellular compartment volume was calculated by multiplying (1−ECV)×(LV mass/1.05). Extracellular compartment volume was calculated by multiplying ECV×(LV mass/1.05).^16^

Tagging analysis was conducted using inTag (v1.0; CREATIS Lab, Lyon, France). Endocardial and epicardial contours were drawn on the short-axis spatial modulation of magnetization sequences using a semiautomated process as reported previously.^13^ Peak LV circumferential strain was measured for the 3 slices.

### Statistical Analysis and Power Calculation

Statistical analysis was performed using IBM SPSS Statistics 22.0 (IBM Corp, Armonk, NY). Continuous variables were expressed as mean±SD or median (interquartile range) depending on normality. Categorical variables were expressed as n (%). Paired data at baseline 1 and 1 month were compared by paired *t* test. When comparing 3 paired groups, ANOVA with repeated measures was used. *P*<0.05 was considered statistically significant.

Receiver operating characteristic analysis was used to determine the diagnostic accuracy baseline imaging parameters to predict regression of LV hypertrophy (>10 g) or cavity dilatation (>10 mL). The diagnostic accuracy is expressed as area under the curve and 95% CI. Optimal sensitivity and specificity were calculated using Youden index. Variables were combined by binary logistic regression. Areas under the curve were compared by using validated methods described by DeLong et al.^17^

The study was powered to detect a 7.5% decrease in indexed intracellular compartment volume after 1 month of detraining. Assuming that baseline indexed intracellular compartment volume would be comparable to low-performance athletes in our previous study, which was 47±6 mL/m^2^, a minimum sample size of 25 athletes would be required (power=0.8; α=0.05).^3^

## Results

Thirty-five athletes agreed to take part in the study between November 2016 and March 2018. One athlete was unable to complete the study because of claustrophobia, 1 withdrew because of possible pregnancy, and 5 withdrew consent after the first scan but before the second scan. The final cohort of 28 included 23 male and 5 female athletes with a median age of 24 (interquartile range, 21–30) years. Twenty-three athletes completed the whole protocol, with 5 athletes withdrawing after their 1-month scan because they had resumed full training. Baseline characteristics and their progression throughout the study are shown in Table 1. There were 31±5 days between the baseline and 1-month scan and 94±10 days between the baseline and 3-month scans. Athletes trained in a wide range of sports including soccer (9), rugby (5), running (4), mixed sports (4), cycling (3), hockey (1), netball (1), and triathlon (1). Before their injury, athletes trained median 7 hours per week (interquartile range, 5–9).

**Table 1.** Clinical Characteristics of Subjects Presented as Mean±SD or Median (Interquartile Range)

	Baseline	1 month Detraining	*P* Value vs Baseline	3 months Detraining	*P* Value vs Baseline	*P* Value vs 1 month
n	28	28		23		
Age, y	24 (21–30)					
Male sex (%)	23 (82)	23 (82)		20 (87)		
Height, cm	177±8					
Weight, kg	78±14	77±12	0.21	76±12	1.0	1.0
Hours per week spent training	7 (5–9)	0 (0–0)	<0.0001	0.5 (0–6)	<0.0001	0.0001
Heart rate	65±10	68±11	0.20	62±9	0.19	0.05
Systolic blood pressure, mm Hg	121 (118–131)	122 (114–130)	0.39	119 (113–124)	0.01	0.17
Diastolic blood pressure, mm Hg	64 (60–75)	65 (57–72)	0.64	63 (54–68)	0.16	0.18

### Changes in Surface ECG

On 1-month detraining, there was a significant decrease in the voltage of the R wave in chest lead V_5_ and the Sokolow-Lyon product, both electrical markers of LV mass (Table 2). There were no significant changes in heart rate, PR interval, or QTc.

**Table 2.** ECG Findings

	Baseline	1 month Detraining	*P* Value vs Baseline	3 months Detraining	*P* Value vs Baseline	*P* Value vs 1 month
Heart rate, bpm	67±11	70±9	0.17	64±8	0.06	0.05
PR interval, s	145±21	148±22	0.13	147±19	0.64	1.0
QTc interval, s	410±19	416±22	0.08	409±21	1.0	0.45
S wave V_2_, mV	14±6	14±6	0.36	14±5	0.52	1.0
R wave V_5_, mV	16±5	14±4	0.006	16±6	1.0	1.0
Sokolow-Lyon product, mV	31±8	29±8	0.01	30±7	0.82	0.52
Incomplete right bundle branch block n (%)	5 (18)	4 (14)		4 (17)		
T wave inversion, n (%)	1 (4)	1 (4)		0 (0)		

### Changes in Ventricular Morphology

**Figure 1.**
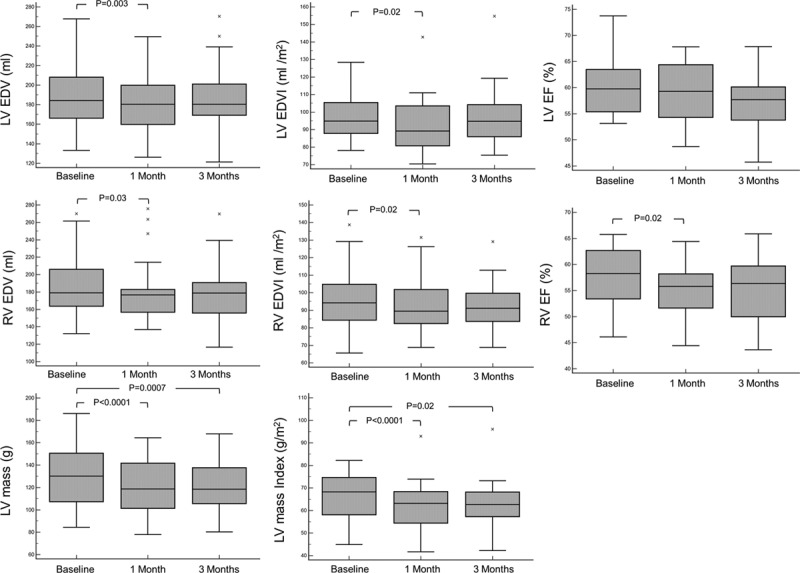
**Change in cardiac morphology on detraining.** Change in left ventricular (LV) and right ventricular (RV) end-diastolic volume (EDV), end-diastolic volume index (EDVI), ejection fraction (EF), and mass after 1 and 3 months of detraining.

**Figure 2.**
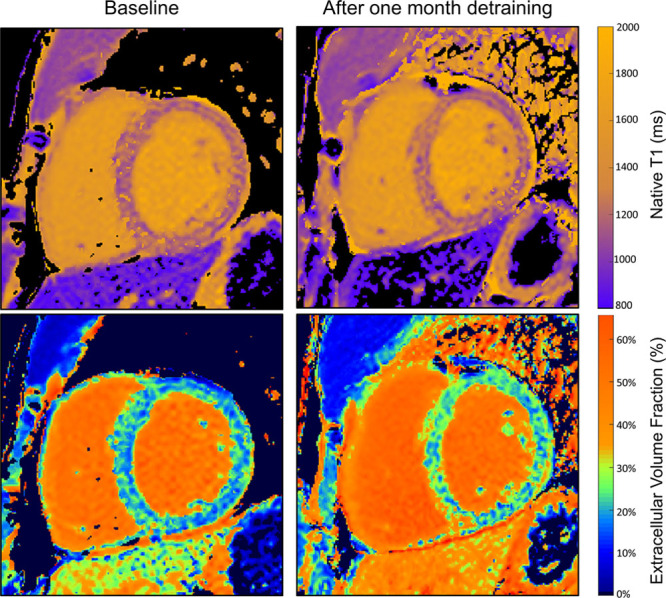
**Native T1 and extracellular volume (ECV) fraction maps before and after 1 month of detraining.** Native T1 (above) and ECV (below) maps from a rugby player before and after 1 month of detraining. During this period, native T1 increased from 1160 to 1213 ms, ECV increased from 19.5% to 23.3%, and left ventricular mass decreased from 186 to 164 g.

**Figure 3.**
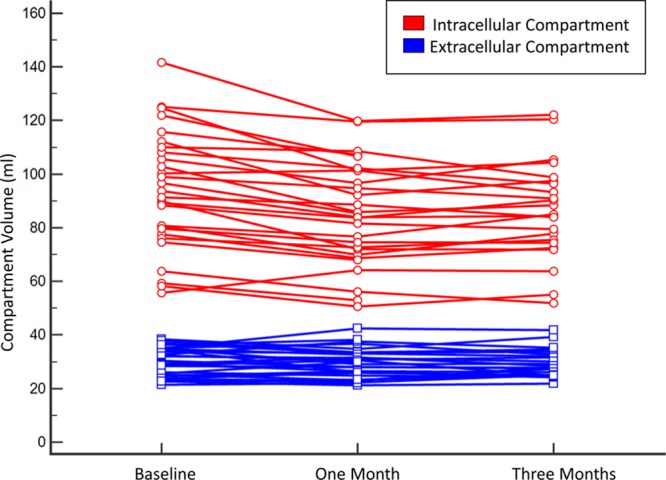
**Change in cardiac compartment volumes on complete detraining.** Individual participant data showing volumes of intracellular (red) and extracellular (blue) compartments at baseline and then after 1 and 3 months of detraining.

After 1 month of complete detraining, there was a 9.3-g (7%; *P*<0.0001) reduction in LV mass with no further reduction between 1 and 3 months (Figure 1; Table 3). This remained significant when indexed to baseline body surface area. In the first month, there were significant increases in native T1 and ECV (Figure 2). There was a decrease in intracellular compartment volume (8.4 mL, 9%; *P*<0.0001) with no significant change in the extracellular compartment mass (Figure 3).

**Table 3.** Cardiovascular Magnetic Resonance Findings

	Baseline	1 month Detraining	*P* Value vs Baseline	3 months Detraining	*P* Value vs Baseline	*P* Value vs 1 month
LV EDV, mL	190±32	182±30	0.003	188±35	1.0	0.38
LV EDV index, mL/m^2^	98±13	94±15	0.007	98±17	1.0	0.34
LV ESV, mL	76±17	75±17	0.72	81±21	0.18	0.37
LV EF, %	60±5	59±5	0.27	57±5	0.07	0.69
LV mass, g	130±28	121±25	<0.0001	121±23	0.0007	1.0
LV mass index, g/m^2^	66±10	62±11	0.0005	63±11	0.02	1.0
RV EDV, mL	188±39	181±35	0.03	180±35	0.59	1.0
RV EDV index, mL/m^2^	97±17	93±15	0.02	93±13	0.53	1.0
RV ESV, mL	80±18	81±15	0.76	81±19	1.0	1.0
RV EF, %	57±6	55±5	0.02	55±7	0.51	1.0
Native T1, ms	1225±30	1239±30	0.02	1228±47	1.0	0.79
ECV, %	24.5±2.3	26.0±2.6	0.0007	25.6±2.8	0.04	1.0
Extracellular compartment volume, mL	30±5	30±5	0.49	29±5	1.0	1.0
Intracellular compartment volume, mL	94±22	85±19	<0.0001	86±18	0.0002	1.0
Circumferential strain: apex, %	12.8±4.5	13.2±3.1	0.61	14.0±3.2	0.57	0.32
Circumferential strain: mid LV, %	13.4±4.5	14.5±2.7	0.30	14.1±2.3	1.0	1.0
Circumferential strain: base, %	13.0±4.1	14.4±2.6	0.06	14.1±2.9	0.45	1.0

ECV indicates extracellular volume fraction; EDV, end-diastolic volume; EF, ejection fraction; ESV, end-systolic volume; LV, left ventricle; and RV, right ventricle.

After 1 month of complete detraining, there were significant comparable decreases in the EDV of both ventricles (ΔLV: −8.2 mL, −4.3%, *P*=0.003; ΔRV: −7.8 mL, −4.1%, *P*=0.03). By 3 months of detraining, there was no further decrease in EDV of either ventricle (Table 3). There was no difference in these temporal changes when they were indexed to baseline body surface area. There was no significant change in LV ejection fraction throughout detraining, but there was a reduction in RV ejection fraction after 1 month because of decreased RV EDV.

No athlete had scarring detected on LGE imaging on any scan.

After 1 month of detraining, there were nonsignificant absolute increases in peak circumferential strain in all 3 levels (Δapex: 0.4%, *P*=0.61; Δmid LV: 1.1%, *P*=0.30; Δbase: 1.4%, *P*=0.06). There were no further changes at 3 months.

### Comparison of Those Who Had and Who Had Not Resumed Training

Between the 1- and 3-month scan, 11 of 23 athletes were able to restart light training but were still not able to resume full training. When athletes were split according to those who had resumed light training (n=11) and those who had not (n=12), there was no difference in any LV or RV volumetric parameter, native T1, or ECV (Table I in the Data Supplement).

### Baseline Parameters to Predict Cardiac Regression

High LV mass index, low native T1, and low ECV at baseline were all predictive of an absolute LV mass regression in 1 month of detraining of >10 g (*P*=0.0006, 0.04, and 0.03, respectively; Table II in the Data Supplement). The difference in diagnostic accuracy between LV mass index and native T1 (*P*=0.58) or ECV (*P*=0.71) was not significant. When LV mass index was combined with either native T1 or ECV by a binary logistic model, there was an improvement in diagnostic accuracy (Table II in the Data Supplement). None of LV EDV index, native T1, or ECV were predictive of absolute LV EDV regression in 1 month of detraining of >10 mL. Only RV EDV index, but not native T1 or ECV, was predictive of absolute RV EDV regression in 1 month of detraining of >10 mL.

## Discussion

We have shown that in athletes after just 1 month of complete detraining, there is regression of LV mass, LV EDV, and RV EDV. There was no further regression of any measure by 3 months of detraining. The regression of LV mass is mediated by a decrease in intracellular compartment volume (predominantly cardiac myocytes) with no change in extracellular compartment volume. High baseline LV mass is the strongest predictor of regression of LV hypertrophy after 1 month of detraining. Low native T1/ECV was also predictive of LV mass regression at 1 month and may have a role in the diagnosis of athlete’s heart.

### Insights Into the Mechanisms of Athletic Ventricular Remodeling

We have demonstrated that regression in LV mass is mediated by a decrease in the intracellular myocardial compartment with no change in the size of extracellular compartment. Previous studies have demonstrated that athletes have lower ECV than sedentary controls and that the fittest athletes have the lowest ECV.^3,4^ These studies were cross-sectional and, therefore, cannot be used to attribute causality. Our present study is the first to show a longitudinal relationship between LV mass, ECV, and training, confirming the hypothesis that athletic hypertrophy is mediated by an increase in the cellular compartment.

When T1 mapping data and LV mass are combined, it is possible to dichotomize the myocardium into cellular and extracellular compartments. This pattern is particularly relevant in hypertrophic phenotypes and has been validated most comprehensively in aortic stenosis; where the derived extracellular compartment volume has a strong correlation with diffuse fibrosis on biopsy^18^ and there is regression of both the cellular and extracellular compartments after aortic valve replacement.^16^

The most important differential diagnosis in the young athlete with LV hypertrophy is HCM. CMR tissue characterization has been histologically validated in HCM and can be used to detect both diffuse fibrosis (increased ECV) and replacement fibrosis (focal LGE).^19,20^ High-level athletes with HCM are reported to have an altered phenotype with more prominent cavity dilatation, but replacement fibrosis is still identified in 33%.^21^ In HCM, focal fibrosis is progressive, and the extent of LGE increases progressively during the course of the disease.^22^ Saberi et al^23^ performed a study of 113 patients with HCM who were randomized to a 16-week programme of moderate-intensity exercise or standard care. They reported that exercise training led to increased exercise capacity but did not change ventricular volumes or the extent of focal fibrosis on LGE.

It would be appealing to conduct a study of detraining in high-level athletes with HCM to investigate reversibility of changes in cellular and extracellular compartments. However, in practice, such a study would be almost impossible given the small number of patients with HCM who participate in competitive sport and their low willingness to voluntarily detrain.

T1 mapping in athlete’s heart has not been validated histologically. However, there are preclinical rat models of exercise-induced cardiac hypertrophy, which suggest physiological hypertrophy is not mediated by increase in myocardial collagen.^6,7^ Benito et al^24^ trained rats to run on a treadmill for an hour a day. After 8 weeks of training, there was an 11% increase in LV mass but no alteration in the hydoxyproline (a modified amino acid found specifically in collagen) content of the whole LV. These results are in keeping with our finding that athletic hypertrophy is mediated by preferential expansion of the intracellular compartment.

### Effects of Detraining on Cardiac Morphology

Previous studies have demonstrated that the heart is highly adaptable to physical training, and cross-sectional studies have clearly demonstrated a dose-response relationship between degree of fitness (measured quantitatively by cardiopulmonary exercise test) and extent of LV and RV remodeling.^25^ Arbab-Zadeh et al^26^ demonstrated in a longitudinal study of 12 previously sedentary subjects when trained for endurance sport development of cardiac athletic remodeling, albeit to a lesser extent than that seen in elite athletes. They reported that in the first 3 months of training, there were significant increases in RV EDV and LV mass with increase in LV EDV by 6 months. Most of the subjects were training for the marathon, and it is not known whether these longitudinal patterns of remodeling apply to other sports or training regimes. Cardiac hypertrophy regression with detraining in our study was quicker, taking only 1 month, compared with remodeling on commencement of training, which took at least 3 months in the previous study.^26^ Although it should be noted that the previous study did not conduct imaging at 1 month and participants in both studies conducted different sports with different baseline fitness.

The evidence of regression of athletic ventricular remodeling with detraining largely predates CMR, and studies were conducted by echocardiography. Maron et al^9^ reported that LV mass measured by echocardiography in 6 Olympic rowers/canoeists decreased by 75 g (24%) in a voluntary period (mean, 13 weeks) of detraining following the 1988 Seoul Olympic Games. Weiner et al^10^ reported a significant regression in LV mass of 4 college American Football players with LV hypertrophy after 3 months of voluntary detraining that returned to pretraining level by 6 months. In a cohort of 40 Olympians, Pellicia et al^8^ reported a 28% reduction in LV mass after long-term detraining (1–13 years). These studies included athletes with LV hypertrophy at baseline (>12 mm interventricular septum) and included athletes at the pinnacle of fitness. The LV hypertrophy was more pronounced in these studies than ours, reflecting the fitness of the athletes studied. The extent of LV mass regression was, therefore, greater (24%–28% versus 7%). These studies defined detraining as reduction in exercise intensity rather than complete cessation, perhaps explaining why regression of LV mass not reported until 3 months.

Pedlar et al^27^ performed echocardiography in 21 amateur runners after an 18-week training programme and then after 4 and 8 weeks when participants were limited to <2 hours of training a week. Similar to our findings, they reported a 10.4% reduction in LV mass after 4 weeks with no change in LV EDV even 8 weeks post-race.

The finding of early regression of LV mass is not unique to athletes and has been reported in by CMR in healthy individuals (n=5) after 6 weeks of complete voluntary bed rest^28^ and by echocardiography in astronauts (n=38) immediately after a 9- to 16-day spaceflight.^29^

The mean LV mass in the present study (130±28 g) was comparable to low-performance male athletes in our previous study (129±17 g) who had a mean VO_2max_ of 60±8 mL/kg per min. If we had been able to recruit higher performance athletes with higher LV mass at baseline, we may have been able to detect a further decrease in LV mass between 1 and 3 months. An alternative explanation is that the pattern of regression reflects the nature of detraining. Athletes were most incapacitated immediately after their fracture leading to most regression in this period. Throughout the subsequent recovery, the levels of physical activity gradually increased affecting the regression response.

Using the same CMR tagging methodology, we have previously shown that athletes have lower peak circumferential strain than sedentary controls.^13^ In the current study, we found that in all 3 levels, there was a no significant increase in strain on detraining, despite significant decreases in LV mass and intracellular compartment in the same period.

### Limitations

In this study, we relied on self-reported abstinence from training, and it is, therefore, possible that athletes performed training that was not reported to the research team. We have not conducted an objective assessment of fitness using cardiopulmonary exercise test, but this was not possible because of the nature of the participants’ injuries. Athletes in this study participated in a range of sports giving different patterns of athletic remodeling at baseline. We did not collect data on nonsteroidal anti-inflammatory use, which may have caused fluid retention and altered the myocardial extracellular compartment. We have not studied athletes with an abnormal ECG, overt LV hypertrophy (12–15 mm), or cardiomyopathy, and patterns of regression in these groups remain to be established.

T1 mapping has only been validated histologically in disease and is difficult to validate in athlete’s heart. Native T1 (and less so ECV) varies by field strength, manufacturer, and pulse sequence. At present, it is recommended that normal values specific to the scanner and acquisition protocol are used to determine ECV in the athlete with unexplained LV hypertrophy.^30^

### Conclusions

Regression of athletic LV hypertrophy can be detected after just 1 month of complete detraining and is mediated by a decrease in the intracellular myocardial compartment with no change in the extracellular compartment. Further studies are needed in athletes with overt and pathological hypertrophy to establish whether native T1 and ECV may complement electrocardiography, echocardiography, cardiopulmonary exercise testing, and genetic testing in predicting the outcome of detraining.

## Supplementary Material

SUPPLEMENTARY MATERIAL
