# Supplementary material for: Regression of Left Ventricular Mass in Athletes Undergoing Complete Detraining Is Mediated by Decrease in Intracellular but Not Extracellular Compartments
Source: Circ Cardiovasc Imaging. 2019 Sep 11;12(9):e009417. doi: 10.1161/CIRCIMAGING.119.009417 (PMC7099858; doi:10.1161/CIRCIMAGING.119.009417)
Supplement: SUPPLEMENTARY MATERIAL [file circimaging-12-e009417-s001.pdf]

SUPPLEMENTAL TABLES

Supplemental Table 1. Comparison between one month and three month scans according to whether athletes had restarted training

	Not yet restarted training			Had resumed light training		
	**One month**	**Three months**	***P value***	**One month**	**Three months**	***P value***
N	12	12		11	11	
LV EDV (ml)	179 ± 30	181 ± 33	*0.56*	190 ± 34	196 ± 36	*0.11*
LV EDV index (ml/m^2)	91 ± 11	93 ± 14	*0.51*	100 ± 18	103 ± 20	*0.11*
LV ESV (ml)	71 ± 15	74 ± 17	*0.31*	82 ± 18	87 ± 24	*0.27*
LV EF (%)	60 ± 6	59 ± 5	*0.34*	57 ± 5	56 ± 5	*0.48*
LV mass (g)	117 ± 25	118 ± 22	*0.82*	125 ± 24	126 ± 24	*0.65*
LV mass index (g/m^2)	60 ± 10	60 ± 9	*0.71*	65 ± 12	66 ± 12	*0.73*
RV EDV (ml)	179 ± 37	177 ± 29	*0.74*	184 ± 40	183 ± 41	*0.88*
RV EDV index (ml/m^2)	93 ± 14	92 ± 11	*0.80*	95 ± 16	94 ± 16	*0.80*
RV ESV (ml)	80 ± 15	76 ± 16	*0.20*	81 ± 16	85 ± 22	*0.22*
RV EF (%)	55 ± 4	57 ± 5	*0.25*	56 ± 5	53 ± 8	*0.19*
Native T1 (ms)	1228 ± 26	1217 ± 59	*0.39*	1224 ± 31	1239 ± 26	*0.50*
ECV (%)	26.4 ± 2.8	26.5 ± 3.3	*0.87*	25.1 ± 2.1	24.7 ± 1.7	*0.42*

Supplemental Table 2: Models to predict regression of left ventricular hypertrophy and cavity dilatation

	AUC	95% CI	P value	Optimal Cut-off	Sensitivity	Specificity
			**Regression in LV mass ≥10g**			
**Baseline LV mass index**	0.81	0.62 - 0.93	0.0006	>72g/m^2	72.7%	88.2%
**Baseline Native T1**	0.73	0.53 - 0.88	0.04	<1207ms	55%	94%
**Baseline ECV**	0.75	0.56 - 0.90	0.03	<23.2%	72.7%	88.2%
**LV mass index + native T1**	0.85	0.66 - 0.95	<0.0001			
**LV mass index + ECV**	0.82	0.63 - 0.94	0.0001			
			**Regression in LV EDV ≥10ml**			
**Baseline LV EDV index**	0.68	0.48 - 0.84	0.11			
**Baseline Native T1**	0.58	0.38 - 0.76	0.48			
**Baseline ECV**	0.52	0.33 - 0.71	0.86			
			**Regression in RV EDV ≥10ml**			
**Baseline RV EDV index**	0.73	0.53 - 0.88	0.02	>101ml	67%	79%
**Baseline Native T1**	0.54	0.34 - 0.73	0.74			
**Baseline ECV**	0.59	0.39 - 0.77	0.43			
